# Treatment of Ulcers

**Published:** 1853-08

**Authors:** 


From the Southern Medical and Surgical Journal.

*Treatment of Ulcers.*—We find in the "Revue de Therapeutique, &c.," that Dr. Lasanna, an Italian physician, recommends highly an ointment made of equal parts of tincture of Iodine and Lard in the treatment of ulcers in general. The application is made twice a day, and has, according to him, healed in ten days most obstinate ulcers.

